# A Dexterous Reorientation Strategy for Precision Picking of Large Thin Objects †

**DOI:** 10.3390/s25206496

**Published:** 2025-10-21

**Authors:** Jungwon Seo

**Affiliations:** Department of Electric and Electronic Engineering, Pusan National University, Busan 46241, Republic of Korea; junseo.kr@pusan.ac.kr

**Keywords:** grasping, dexterous manipulation, grippers and end-effectors, force sensing

## Abstract

This paper presents tilt-and-pivot manipulation, a robotic technique for picking large, thin objects resting on hard supporting surfaces. The method employs in-hand dexterous manipulation by reorienting the gripper around the object’s contact point, allowing a finger to enter the gap between the object and the surface, without requiring relative sliding at the contact. This finally facilitates reliable pinch grasps on the object’s faces. We investigate the kinematic principles and planning strategies underlying tilt-and-pivot, discuss effector design considerations, and highlight the practical advantages of the strategy, which is applicable to a variety of low-profile objects. Experimental results, incorporating vision and force–torque sensing, demonstrate its effectiveness in bin-picking scenarios and its applicability to more complex object-handling tasks.

## 1. Introduction

**Figure 1:**
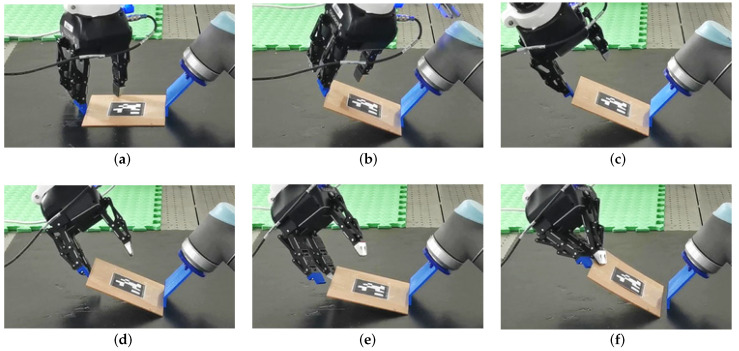
Progression of tilt-and-pivot. (**a**,**b**) The object is tilted upward, making contact with the gripper and a fixture on the right. (**c**,**d**) The gripper pivots about the contact, inserting another finger beneath the object. (**e**,**f**) The sequence concludes with a secure pinch grasp. Reprinted with permission from [1].

This paper investigates a robotic object handling strategy termed tilt-and-pivot manipulation, tailored for rigid low-profile objects with small thickness and primarily aimed at picking them from supporting surfaces (This article is a revised and expanded version of [1], which was presented at the 2020 IEEE International Conference on Robotics and Automation (ICRA), Paris, France, May 2020). The method involves dexterous manipulation, with the configuration of the effector (or gripper) relative to the object changing over time. Figure 1 illustrates the execution of the technique using a dual-arm manipulation setup equipped with a three-fingered gripper. In the tilt phase, the robot rotates the object upward with one finger of the gripper while using the other arm as a supporting axis. In the pivot phase, the gripper reorients by pivoting around the established contact, enabling another finger to enter the space between the object and the ground surface. Closing the fingers then yields a secure pinch grasp.

Handling thin objects is a key practical capability in robotics, relevant to both industrial and service applications. Nevertheless, their small thickness makes them inherently challenging to grasp. The difficulty is further compounded even by commonplace obstacles such as hard supporting surfaces—for example, lifting a plastic card from a tabletop. Conventional robotic systems with standard two- or three-fingered grippers often face limitations in this task due to their restricted dexterity.

The presented tilt-and-pivot technique provides a promising solution to these challenges. We demonstrate its feasibility by identifying achievable target configurations, outlining key design principles for the effector, and presenting a method for reliably executing the motion toward the goal. The approach is shown to be compatible with standard robotic platforms, such as motion-controlled arms, multi-fingered grippers, and wrist force–torque sensors. Experimental validation is conducted in bin-picking tasks, where individual objects—not constrained by the workspace size of the gripper—in random poses are sequentially retrieved from clutter. For autonomous operation, the method combines vision with force–torque sensing. Furthermore, we demonstrate how successful picking by tilt-and-pivot can serve as a foundation for subsequent object-handling tasks, enabling more complex manipulation.

The remainder of the paper is organized as follows. Relevant literature is outlined in Section 2, and Section 3 formally defines the research problem. Section 4 covers grasp planning, specifying the desired target configurations. Section 5 focuses on effector design. Section 6 details the steps of the tilt-and-pivot manipulation. Section 7 discusses the use of multi-fingered grippers for tilt-and-pivot. Section 8 presents experimental results, demonstrating applications to bin picking and more complex tasks. In particular, the key advancements beyond the prior conference version [1] include a revised discussion on manipulation execution (Section 6), an expanded treatment of multi-fingered grippers with a new proof of Theorem 1 (Section 7), and additional experimental results (Section 8).

## 2. Related Work

Robotic manipulation of thin objects, the focus of this paper, has received considerable attention due to its practical importance. For instance, ref. [2] proposes an open-loop picking strategy using an underactuated hand and demonstrates successful handling of various thin objects. Ref. [3] introduces a picking strategy that exploits the deformation of soft fingertips. Ref. [4] presents a gripper design and associated manipulation methods suitable for scooping thin objects resting on a flat surface. Our earlier studies explored dynamic manipulation for thin, flexible objects [5] and quasi-static methods for small (relative to the gripper workspace), thin parts [6]. In contrast, this paper addresses large (relative to the gripper workspace), rigid parts, thereby complementing those prior approaches.

Our object picking approach falls under the category of robotic in-hand manipulation, focusing on adjusting the position or orientation of an object within the robot’s hand. This topic has been extensively studied in connection with the broader challenge of robotic dexterity. Examples commonly studied in the literature include finger gaiting, sliding, rolling, and regrasping [7,8,9,10,11] (see also references therein), typically demonstrated with multi-fingered grippers. More recently, researchers have explored in-hand manipulation in combination with environmental interactions. For example, refs. [12,13,14] propose methods that make use of external forces and environmental constraints. Design-focused strategies, including the use of mechanical compliance and underactuation, have been explored in [15,16]. From a planning perspective, ref. [17] introduces an optimization-based approach for planning scooping motions that involve contact with external surfaces. Our earlier studies [18,19] also demonstrated that conventional grippers can perform environment-assisted in-hand manipulations, enabling placement operations through sliding and rolling. The present work extends this repertoire by introducing pivoting as a distinct manipulation capability.

Robotic bin picking has been an active research area, with direct relevance to a wide range of practical applications. A key requirement for successful bin picking is object recognition, where vision-based methods—often combined with solid object models—have been widely adopted [20,21,22,23]. Another critical component is manipulation for picking. In many industrial solutions, specialized end-effectors such as suction grippers are commonly used, despite their limitations. Recently, learning-based approaches have shown strong potential to enhance bin picking performance. For example, ref. [24] presents a learning-based grasp detection method that facilitates successful picking from dense clutter, while [25] demonstrates the use of deep neural networks trained on RGB-D images for end-to-end bin picking. Additional examples of this approach can be found in [26,27]. Finally, bin packing tasks can be regarded as the dual problem to bin picking. For instance, ref. [28] proposes an end-to-end bin packing solution that incorporates manipulation primitives to compensate for errors and uncertainties.

## 3. Problem Description

**Figure 2:**
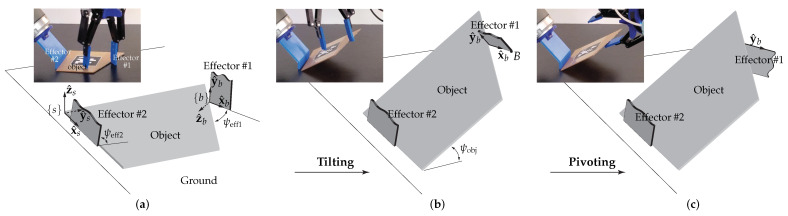
Progression of a tilt-and-pivot operation for a trapezoidal polygonal object using rectangular Effectors #1 and #2: (**a**,**b**) tilting and (**b**,**c**) pivoting. The fixed space frame is denoted by {s}, and the body frame {b} is attached to the corner vertex of Effector #1 currently in point contact with the object. In (**a**), the ${\hat{x}}_{b}{\hat{y}}_{b}$-plane (Effector #1’s plane) is perpendicular to the ${\hat{x}}_{s}{\hat{y}}_{s}$-plane (the object or ground plane); the ${\hat{x}}_{b}$-axis and the object edge form the angle $\psi_{\mathrm{eff}1}$, while Effector #2 makes an angle $\psi_{\mathrm{eff}2}$ with the ground. In (**b**), the object is tilted upward by $\psi_{\mathrm{obj}}$, while the relative configuration between the object and Effector #1, as well as $\psi_{\mathrm{eff}2}$, remain unchanged. Finally, in (**c**), Effector #1 pivots about the contact, placing its other corner vertex *B*, shown in (**b**), between the object and the ground. Both $\psi_{\mathrm{obj}}$ and $\psi_{\mathrm{eff}2}$ remain unchanged. Insets show corresponding photographs from real experiments. Reprinted with permission from [1].

This paper investigates the robotic task of picking low-profile objects with minimal thickness from supporting surfaces such as tabletops—a frequently encountered scenario in practical bin-picking applications. As illustrated in Figure 2, the object of interest is modeled as a rigid polygonal plate with negligible thickness, resting on a rigid horizontal ground surface. We specifically consider large objects whose dimensions exceed the gripper’s maximum opening range, rendering conventional parallel-jaw grasping across the object’s face infeasible.

We consider a minimal robotic setup with simple effectors, each abstracted as a single rigid body without internal mobility, as shown in Figure 2. We assume all contacts are unilateral and accordingly transmit pushing forces only; bilateral suction-based gripping is not considered under this assumption. We adopt these conservative assumptions to guarantee practicality and robustness in the solution.

Transitioning from the initial configuration—where the object is placed on the ground—to a target configuration—where it is securely grasped by the robot—often requires dexterous manipulation involving reconfiguration of the effectors relative to the object. One existing approach is scooping manipulation, as demonstrated in [4], which relies on sophisticated hardware (e.g., retractable nails and epicyclic mechanisms) and coordinated arm-gripper motion planning. In contrast, this work investigates an approach designed for simpler, conventional hardware such as a motion-controlled parallel-jaw gripper and a wrist force–torque sensor, while maintaining sufficient generality to handle any rigid polygonal object. This formulation not only captures a broad class of practical objects but also enables seamless integration with additional manipulation tasks beyond picking, all within a simple robotic setup.

## 4. Establishing Target Configurations

Our proposed strategy, termed tilt-and-pivot manipulation, consists of two key steps. First, the object resting on the surface is tilted to create an opportunity for picking. Second, the effector is guided into the gap formed between the object and the surface by executing a three-dimensional pivoting motion. In this section, we discuss the desired target configuration of the effector–object system. The target configuration is specified by the (1) contact locations between the object and effectors, as well as (2) the overall orientation of the effector–object system.

### 4.1. Locating Target Contacts

As illustrated in Figure 2a, the object is engaged by two rectangular effectors. Effector #1 is intended to establish a point contact with the object, either at one of its vertices or along an edge. This contact occurs at a vertex of Effector #1, which also defines the origin of the body frame {b} attached to Effector #1. However, such a contact can be unstable, since even slight misalignment may cause the contact to break. A solution to this problem, involving the use of concave contact surfaces, is discussed in Section 5. Effector #2 establishes contact with the supporting surface through its bottom edge, while the corner formed at this interface engages an edge of the object, resulting in a line contact.

The resulting element pair of the object on which the contacts are formed—〈vertex, edge〉 or 〈edge, edge〉 (Figure 2 shows an 〈edge, edge〉 pair)—are chosen, along with their specific locations along the edges, to achieve force-closure [29], thereby securely constraining the object against external disturbances. From a computational standpoint, this problem reduces to searching for planar force-closure grasps on the object plane, as the effector design inherently restricts motion out of the plane (this issue is revisited in Section 5). In our earlier work [30], we presented a method for locating 〈vertex, edge〉 contacts that guarantee form-closure, a condition stricter than force-closure. For grasps of type 〈edge, edge〉, the procedure is as follows: (1) approximate the line contact at Effector #2 as two frictional point contacts—resulting in three point contacts in total, including that of Effector #1—and (2) verify force-closure via linear optimization. This strategy enables grasping of arbitrary rigid polygonal shapes in force-closure [30].

For convex objects, these grasps are inherently collision-free, since the effectors cannot interfere with other parts of the convex hull. The search procedure may yield multiple feasible solutions. In such cases, the element pair with the shortest distance between the point (at Effector #1) and line (at Effector #2) contacts is chosen, for reasons explained in Section 6.1.

### 4.2. Target Orientation

The desired target orientation of the effector–object system is specified as an element of $R^{2}\times SO\left(3\right)$, consisting of $\psi_{\mathrm{obj}}\in R$ (the angle between the object and the ground, Figure 2b), $\psi_{\mathrm{eff}2}\in R$ (the angle between Effector #2 and the ground, Figure 2a), and R∈SO(3) (the orientation of Effector #1).

First, the matrix R is chosen to satisfy the following criteria:

- The target orientation of the ${\hat{x}}_{b}$-axis of the body frame {b}, denoted ${\hat{x}}_{b}^{\prime }$, is fixed such that vertex *B* (Figure 2b) of Effector #1 lies within the interior of the solid of revolution generated by rotating the object around the axis aligned with the ${\hat{x}}_{s}$-axis of the space frame {s}. See Figure 2c. This condition ensures that the object remains supported, not falling to the ground, even if the pivot contact is disengaged. Fixing ${\hat{x}}_{b}^{\prime }$ determines two of the three independent parameters of R.
- The remaining third parameter of R, representing the relative orientation of Effector #1 about the ${\hat{x}}_{b}^{\prime }$-axis, is chosen to ensure that Effector #1 is not in collision with the object (Figure 2c).

Next, $\psi_{\mathrm{obj}}$ is chosen such that $\psi_{\mathrm{obj}}<90^{\circ }$ and Effector #1 avoids collision with the ground. The angle $\psi_{\mathrm{eff}2}$ is then selected to satisfy $\psi_{\mathrm{obj}}<\psi_{\mathrm{eff}2}<90^{\circ }$, ensuring that the object remains positioned between Effector #2 and the ground.

We represent R using YXZ Euler angles, denoted (α,β,γ), which define the orientation of {b} relative to its initial configuration. In this reference configuration, the angle $\psi_{\mathrm{eff}1}$—between ${\hat{x}}_{b}$ and the object edge in contact with Effector #1 (see Figure 2a)—is set to zero, and ${\hat{y}}_{b}$ is perpendicular to the object plane. Throughout this work, we impose the bounds $|\alpha |,|\beta |,|\gamma |<\frac{\pi }{2}$. These constraints serve two purposes: they shorten the manipulation duration and facilitate both the design of effector geometries (Section 5) and collision avoidance in multi-fingered gripper setups (Section 7).

A target orientation for Effector #1 can be defined while respecting the bounds on α, β, and γ. For instance, setting β very close to $-\frac{\pi }{2}$ produces a configuration near gimbal lock, where the rotation axes for α and γ become nearly colinear. Given $0<\alpha <\frac{\pi }{2}$, which initially points ${\hat{x}}_{b}$ away from the solid of revolution, it is possible to choose γ such that $0<|\alpha |<|\gamma |<\frac{\pi }{2}$ and γ<0, thereby counteracting the rotation due to α and guiding ${\hat{x}}_{b}$ into the interior of the solid of revolution. The existence of feasible paths leading to such target configuration is further discussed in Section 7.

**Figure 3:**
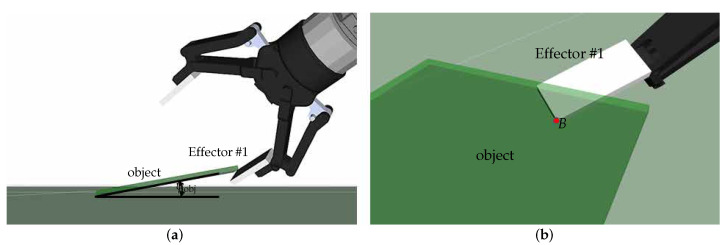
(**a**) A feasible goal configuration for Effector #1, illustrated as part of a two-fingered gripper, with $\psi_{\mathrm{obj}}=10^{\circ }$, $\alpha =20^{\circ }$, $\beta =-50^{\circ }$, and $\gamma =-60^{\circ }$. (**b**) Top view of the same configuration. Vertex *B* is tucked between the object and the ground, as in Figure 2c. Reprinted with permission from [1].

The search for feasible target configurations was performed using a sampling-based approach with collision checking implemented via the ROS MoveIt motion planning framework. Figure 3 illustrates an example, confirming that $|\alpha |,|\beta |,|\gamma |<\frac{\pi }{2}$, with α>0 and β,γ<0.

## 5. Effector Design

**Figure 4:**
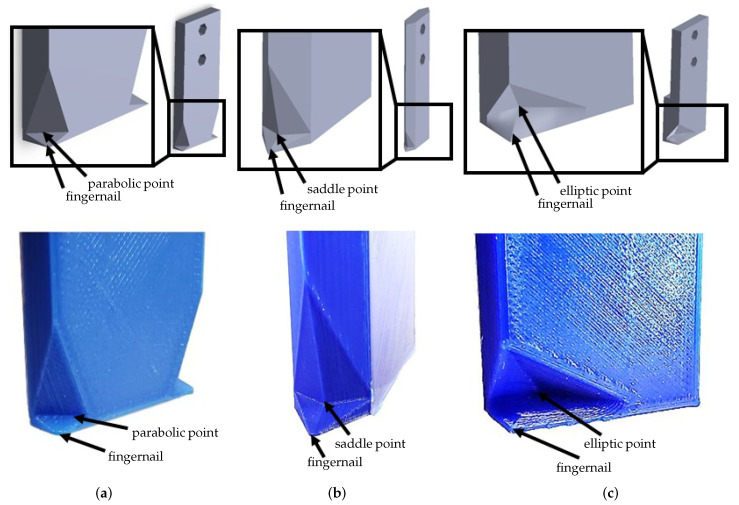
Solid models (**top**) and 3D-printed prototypes (**bottom**) for Effector #1: (**a**) Zero Gaussian curvature design with a parabolic point, (**b**) negative Gaussian curvature design with a saddle point, and (**c**) positive Gaussian curvature design with an elliptic point. Reprinted with permission from [1].

Achieving a reliable point contact between Effector #1 and the object—specifically, the contact at a vertex of the effector with either a vertex or an edge of the object (Figure 2)—is critical. To address the unstable nature of this contact, as noted earlier, and to reliably capture the vertex or edge, the fingertip shape of Effector #1 must be customized with an appropriate concavity that accommodates the target element. Figure 4 illustrates three types of concavities and fingernails at the tip of Effector #1, corresponding to surfaces with zero, negative, and positive Gaussian curvature, respectively. The zero-curvature design is primarily used to capture an object edge via a parabolic point on the effector surface. The negative- and positive-curvature designs are suited for capturing concave and convex object vertices, respectively, using a saddle and an elliptic point on the effector surface. The fingertip concavity, together with the concavity formed on the supporting surface by Effector #2, effectively restricts out-of-plane object motion. This supports the adequacy of planar force-closure for grasp planning outlined in Section 4.1.

The dimensions and geometry of the concavities can be determined based on the trajectories leading to the target configurations. From the fingertip’s perspective, the object sweeps a volume along the motion trajectory. This swept volume determines the portion of material to be removed from the fingertip, guaranteeing that the object stays securely throughout the motion. In this sense, the object itself functions like a carving tool, sculpting the fingertip.

In real applications, effectors may be fabricated in standardized sizes and shapes. The most suitable design can then be selected according to criteria such as the type of element to be contacted and the required motion range. For example, when the effector shown in Figure 4b is used to contact an edge, the allowable range of motion—obtained through kinematic simulation in SolidWorks—is $-82^{\circ }<\gamma \leq 0^{\circ }$ for $\alpha =40^{\circ }$ and $\beta =-65^{\circ }$. This case is demonstrated in our experiments (row 4 of Table 1), where the target (α,β,γ) lies within the feasible motion range. Another criterion is the size of cages permitted by concavity, which is especially relevant in the presence of sensing and positioning errors. Further details on establishing cage configurations, particularly for designs with positive Gaussian curvature, are provided in our prior work [30].

Furthermore, crafting the fingernail to be low-friction and sharp aids the initial physical interaction, as further discussed in Section 6.1. Although this work focuses on rigid-body effectors, passive compliance from soft, flexible materials could also be leveraged to improve grasp stability.

## 6. Execution of Tilt-and-Pivot

This section details the execution of the tilt-and-pivot manipulation strategy.

### 6.1. Making Contacts

At the start, Effectors #1 and #2 are guided to establish contacts with the element pair 〈vertex, edge〉 or 〈edge, edge〉 from the target configuration. For Effector #1, the point of contact must fall within its fingertip concavity. To achieve this, the effectors are first positioned around the object to form a cage configuration and are then moved inward, allowing the fingernail of Effector #1 to slide beneath the object.

Reducing the distance between the point and line contacts eases sliding of the fingernail [4], which supports selecting the element pair with the smallest separation, as described in Section 4.1. Sliding is further facilitated by a sharper, low-friction fingernail [4], as noted in Section 5.

### 6.2. Tilting

Once the contacts are established, Effector #1 tilts the object by rotating it around the line contact, lifting it to the target angle $\psi_{\mathrm{obj}}$ with respect to the supporting surface (Figure 2a,b). The wrist of Effector #1 follows a circular trajectory centered on the axis—the line contact.

### 6.3. Pivoting

Following the tilting phase, the robot executes a pivoting motion, as shown in Figure 2b,c. Effector #1 pivots about the contact point—the origin of {b}—to achieve the target orientation R specified in Section 4.2. Two approaches can be used:

- Matrix logarithm of R: Compute the matrix logarithm of R [29]
  (1) $$\log:R\in SO\left(3\right)\mapsto \left[\hat{w}\right]\theta \in so\left(3\right)$$
  Effector #1 then rotates about the axis defined by the skew-symmetric matrix $\left[\hat{w}\right]$ by the angle θ.
- YXZ Euler angles (α,β,γ) of R: Initially before the tilting phase, set $\psi_{\mathrm{eff}1}=\alpha$ by rotating Effector #1 about ${\hat{y}}_{b}$ by α. Next, rotate about ${\hat{x}}_{b}$ by β (or about the negative ${\hat{x}}_{b}$-axis by |β| since β<0), and finally rotate about ${\hat{z}}_{b}$ by γ (or about the negative ${\hat{z}}_{b}$-axis by |γ| since γ<0).

These maneuvers move the other vertex of Effector #1 (marked *B* in Figure 2b)), initially not in contact, into the interior of the solid of revolution, nested between the object and the ground. This illustrates dexterous manipulation through controlled reorientation of the effector relative to the object.

Although it could be possible to arrange Effector #1 to enter the space by sliding on the edge of the object, initiating relative motion at the contact can be difficult due to the fingertip concavity, uncertain friction, and a large angle of attack (defined as the angle between Effector #1 and the object), close to the right angle. The pivoting strategy circumvents this issue, as it does not require relative sliding motion at the contact.

The fingertip concavity provides caging, enabling the entire tilt-and-pivot sequence to be executed using conventional motion-based control. In contrast, without such caging, maintaining contact at Effector #1 would necessitate hybrid motion–force control, as in levering-up tasks [31].

### 6.4. Aligning

Finally, Effector #1 is reoriented so that its face aligns with the corresponding face of the object, making their normals aligned and allowing the object to rest securely. This alignment is achieved through an additional pivoting motion about vertex *B*. The location of *B* sets the new target orientation: the plane of Effector #1’s face must be aligned to include the line contact at Effector #2. Pivoting around *B*, positioned within the interior of the solid of revolution, ensures that the object remains supported and does not fall.

## 7. Adoption of Multi-Fingered Grippers

As shown in Figure 1 and Figure 2, Effector #1—responsible for generating the tilt-and-pivot motion—can, in practice, be implemented using standard robotic grippers, including two- and three-fingered designs that incorporate additional effectors. The following theorem formalizes that these additional effectors can be configured to guarantee collision-free motion with the object, thereby confirming the suitability of such grippers for this task.

**Figure 5:**
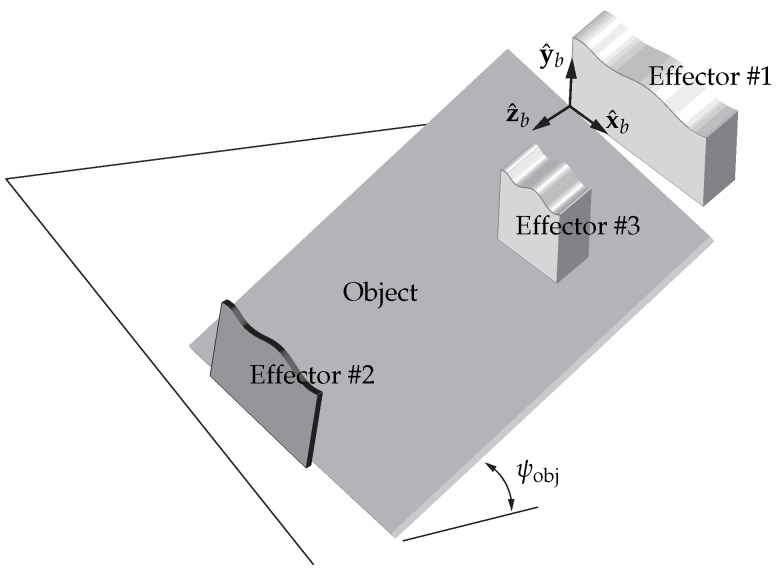
Gripper model with two cuboid-shaped effectors, labeled Effectors #1 and #3, handling a convex polygonal object.

Figure 5 illustrates a representative gripper model to be used in the theorem. It consists of two digits, Effector #1 and the newly added Effector #3, each modeled with a cuboid-shaped collision hull to approximate typical two- and three-fingered grippers with opposing fingers. We assume that the two cuboid-shaped effectors have parallel edges and move as a single rigid body. It is also assumed that Effector #3 is initially collision-free with the object when the gripper’s orientation—represented by the YXZ Euler angles (α,β,γ)—is set to (0,0,0). This is possible if the collision hull of Effector #3 does not extend below the ${\hat{x}}_{b}{\hat{z}}_{b}$-plane. The object of interest is modeled as a convex polygon, as also depicted in Figure 5.

**Theorem** **1.** *The tilt-and-pivot motion can be executed while ensuring that Effector #3 remains collision-free with the object.*

**Proof.**  Consider the YXZ Euler angle representation (α,β,γ) of a feasible target orientation for Effector #1, discussed in Section 4.2. The corresponding rotation matrix R is then given as

(2) $$\begin{array}{cc} R\; & =\mathrm{Rot}\left({\hat{y}}_{b},\alpha \right)\mathrm{Rot}\left({\hat{x}}_{b},\beta \right)\mathrm{Rot}\left({\hat{z}}_{b},\gamma \right) \end{array}$$

(3) $$\begin{array}{cc} \; & =\left[\begin{array}{ccc} \cos\alpha & 0 & \sin\alpha \\ 0 & 1 & 0\\ -\sin\alpha & 0 & \cos\alpha \end{array}\right]\left[\begin{array}{ccc} 1 & 0 & 0\\ 0 & \cos\beta & -\sin\beta \\ 0 & \sin\beta & \cos\beta \end{array}\right]\left[\begin{array}{ccc} \cos\gamma & -\sin\gamma & 0\\ \sin\gamma & \cos\gamma & 0\\ 0 & 0 & 1 \end{array}\right] \end{array}$$

(4) $$\begin{array}{cc} \; & =\left[\begin{array}{ccc} \cos\alpha \cos\gamma +\sin\alpha \sin\beta \sin\gamma & \sin\alpha \sin\beta \cos\gamma -\cos\alpha \sin\gamma & \sin\alpha \cos\beta \\ \cos\beta \sin\gamma & \cos\beta \cos\gamma & -\sin\beta \\ \cos\alpha \sin\beta \sin\gamma -\sin\alpha \cos\gamma & \cos\alpha \sin\beta \cos\gamma +\sin\alpha \sin\gamma & \cos\alpha \cos\beta \end{array}\right] \end{array}$$

Recall the configuration near gimbal lock, also discussed in Section 4.2, where β is set arbitrarily close to $-\frac{\pi }{2}$. R then becomes

(5) $$R=\left[\begin{array}{ccc} \cos\alpha \cos\gamma -\sin\alpha \sin\gamma & -\sin\alpha \cos\gamma -\cos\alpha \sin\gamma & 0\\ 0 & 0 & 1\\ -\cos\alpha \sin\gamma -\sin\alpha \cos\gamma & -\cos\alpha \cos\gamma +\sin\alpha \sin\gamma & 0 \end{array}\right]$$

Now consider the matrix logarithm of R to find its exponential coordinates—the axis and amount of the rotation [29]:

(6) $$\log:R\in SO\left(3\right)\mapsto \left[\hat{w}\right]\theta \in so\left(3\right)$$

where $\left[\hat{w}\right]$, the skew-symmetric matrix representation of the axis of rotation, is given by

(7) $$\left[\hat{w}\right]=\frac{1}{2\sin\theta }\left(R-R^{T}\right)$$

Since $R-R^{T}$ is

(8) $$\begin{array}{cc} \; & \left[\begin{array}{ccc} 0 & -\sin\alpha \cos\gamma -\cos\alpha \sin\gamma & \cos\alpha \sin\gamma +\sin\alpha \cos\gamma \\ \sin\alpha \cos\gamma +\cos\alpha \sin\gamma & 0 & 1+\cos\alpha \cos\gamma -\sin\alpha \sin\gamma \\ -\cos\alpha \sin\gamma -\sin\alpha \cos\gamma & -1-\cos\alpha \cos\gamma +\sin\alpha \sin\gamma & 0 \end{array}\right] \end{array}$$

the axis of rotation $\hat{w}$ is represented as the following vector:

(9) $$\begin{array}{cc} \hat{w} & =\frac{1}{2\sin\theta }\left[\begin{array}{c} -1-\cos\alpha \cos\gamma +\sin\alpha \sin\gamma \\ \cos\alpha \sin\gamma +\sin\alpha \cos\gamma \\ \sin\alpha \cos\gamma +\cos\alpha \sin\gamma \end{array}\right] \end{array}$$

(10) $$\begin{array}{cc} \; & =\frac{1}{2\sin\theta }\left[\begin{array}{c} -1-\cos(\alpha +\gamma )\\ \sin(\alpha +\gamma )\\ \sin(\alpha +\gamma ) \end{array}\right] \end{array}$$

Recall when $\beta \approx -\frac{\pi }{2}$, α and γ can be chosen such that $0<|\alpha |<|\gamma |<\frac{\pi }{2}$ where α>0 and γ<0 (Section 4.2). Accordingly, the sum of α and γ is bounded:

(11) $$-\frac{\pi }{2}<\alpha +\gamma <0$$

In addition, the amount of rotation θ takes a value between 0 and π by definition:

(12) $$\theta =\cos^{-1}\left(\frac{1}{2}\left(\mathrm{tr}R-1\right)\right)\in \left[0,\pi \right)$$

where trR, the trace of R, is given by

trR=cosαcosγ−sinαsinγ=cos(α+γ)

Now let α+γ be chosen sufficiently close to zero. Under this condition, (1) the ${\hat{x}}_{b}$-component of $\hat{w}$ is nonzero and negative, and (2) that ${\hat{x}}_{b}$-component dominates the others since sin(α+γ) vanishes in Equation (9). In other words, the axis of rotation $\hat{w}$ can be positioned arbitrarily close to the object edge where Effector #1 makes contact (see Figure 5). The only possible mode of collision in this case would be Effector #3 touching the opposite side of the object. However, this does not occur because the rotation angle θ approaches at most $\frac{\pi }{2}$ according to Equation (12), when α+γ is chosen sufficiently close to zero. □

Theorem 1 does not preclude the existence of alternative collision-free paths beyond the proposed matrix-logarithm-based approach. In particular, the YXZ Euler angle motion described in our previous work [1] can also be employed. Similarly, other effector shapes or mildly non-convex objects may admit collision-free trajectories. In such cases, feasibility can be verified constructively using a motion planner.

The ability to use multi-fingered grippers, as supported by Theorem 1, is of practical importance. Such grippers not only enable the object to be securely grasped following the aligning phase in Section 6.4, but also facilitate secondary manipulation tasks by exploiting their dexterity, as will be demonstrated in our experiments. Fingertip concavities or fingernails are not expected to significantly impact the overall functionality of multi-fingered grippers, as the primary contact surface of the finger remains largely unchanged.

We note that while Effector #1 can be incorporated into a multi-fingered gripper, Effector #2 may simply be realized by a stationary element of the environment, such as a bin wall. This further enhances the practical viability of the tilt-and-pivot approach.

## 8. Experiments: Bin Picking and Beyond

This section details the realization of the proposed tilt-and-pivot strategy and its experimental validation in application scenarios. We first consider robotic bin-picking, in which objects are retrieved one by one from a cluttered, unordered pile. We then extend the scenario to more complex object-handling tasks that build on this capability.

### 8.1. Robot System

**Figure 6:**
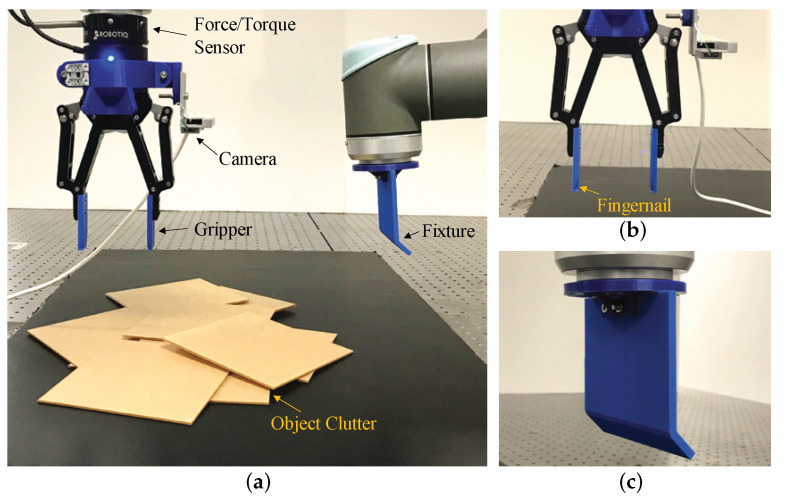
(**a**) Dual-arm robotic platform for bin picking experiments. (**b**) Left-arm gripper customized with a fingertip concavity for tilt-and-pivot manipulation. (**c**) Right-arm effector implemented as a single rigid body to support tilting. Reprinted with permission from [1].

The tilt-and-pivot method is realized on a dual-arm robotic platform, as shown in Figure 6. The setup employs two six-DOF manipulators (UR10; Universal Robots, Denmark): one arm is fitted with a two- or three-fingered gripper (Adaptive Gripper; Robotiq, Canada) to manipulate the object (as Effector #1 in Figure 2), while the other carries a fixture that serves as the tilting axis (Effector #2). Both arms are instrumented with a wrist-mounted force–torque sensor (FT 300; Robotiq, Canada) and a camera for 2D vision (SR300; RealSense, USA).

**Figure 7:**
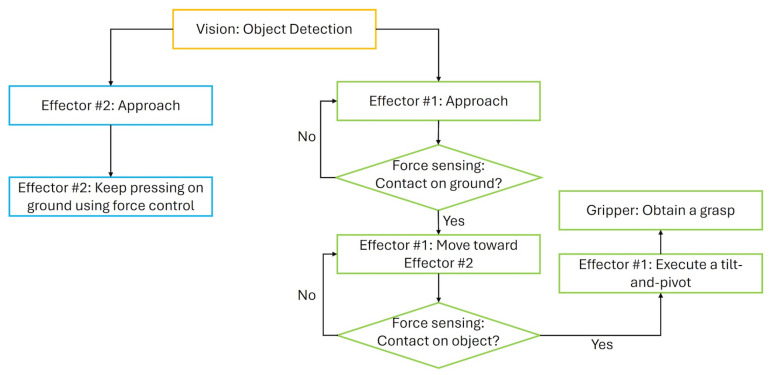
Overview of the software framework integrating vision, force sensing/control, and motion control for tilt-and-pivot picking.

Our software framework (Figure 7) integrates sensing and control functions, including: (1) 2D vision using fiducial markers or instance segmentation for object detection, (2) force–torque sensing to monitor contact events, (3) force control to regulate interaction with the supporting surface, and (4) coordinated motion planning and execution for the dual-armed system to perform the tilt-and-pivot pick. The roles of these modules are elaborated in the description of the experimental procedures that follow. In particular, the adoption of a conventional motion control scheme for Effector #1—enabled by the fingertip concavity (Section 5), which eliminates the need for contact state monitoring and maintenance for the pivoting contact—facilitates the overall implementation.

### 8.2. Experiments

We conducted a series of experiments to validate the proposed approach, encompassing both diverse picking scenarios and secondary manipulation tasks performed after the objects were picked.

#### 8.2.1. Single Object Picking

The proposed picking method was initially evaluated with individual objects placed on a cardboard surface. The test objects comprised a variety of low-profile, flat items of differing sizes and materials, as summarized in Table 1. Each object was placed in a random pose, which was detected using fiducial markers (AprilTag). The effectors were first commanded to move downward toward the object along its footprint, which was intentionally inflated to compensate for sensing errors from AprilTag detection. The descent was halted once the normal force measured by the wrist force–torque sensor exceeded a predefined threshold. Subsequently, the effectors were moved laterally toward each other to establish the desired contact configuration, with force–torque sensing used again to terminate motion upon contact. Finally, a tilt-and-pivot motion was executed in open loop, after which the gripper was closed to securely grasp the object. This sequence is illustrated in Figure 7.

**Table 1:** Experimental results for single object picking. Related results are also reported in [32].

Object/Task	Fingertip Curvature	Object Dimensions (mm)	Target Parameters					Average Duration (s)	Succes sRate
		Length × Width × Thickness	$\psi_{\mathrm{obj}}(\circ )$	$\psi_{\mathrm{eff}2}(\circ )$	α(∘)	β(∘)	γ(∘)		
Single objects ^1^ (Figure 8a)	0	170 × 170 × 2.0	25	85	30	−70	−70	12	215/250
Acrylic board: concave vertex (Figure 8b)	−	220 × 220 × 2.0	23	88	10	−50	−60	12	18/20
Acrylic board: equilat. triangle (Figure 8c)	+	170 (each edge) × 2.0	23	88	50	−70	−80	13	18/20
Acrylic board	−	170 × 170 × 2.0	23	88	40	−65	−70	12	17/20
Acrylic board: expedited	0	170 × 170 × 2.0	23	85	30	−65	−65	4	24/30
Acrylic board: 3-fingered gripper	0	170 × 170 × 2.0	23	88	20	−45	−65	12	17/20
Carton flat	0	265 × 210 × 1.5	35	88	30	−60	−60	12	16/20
Textbook	0	235 × 175 × 20.0	15	87	45	−55	−70	14	15/20
Picture frame picking	0	178 × 127 × 2.0	25	88	45	−35	−50	13	17/20
Container lid opening	0	380 × 260 × 8.0	15	87	35	−60	−50	13	16/20

^1^ The objects tested include acrylic boards, PCBs, plastic boards, and aluminum boards. The dimensions reported correspond to the acrylic board.

**Figure 8:**
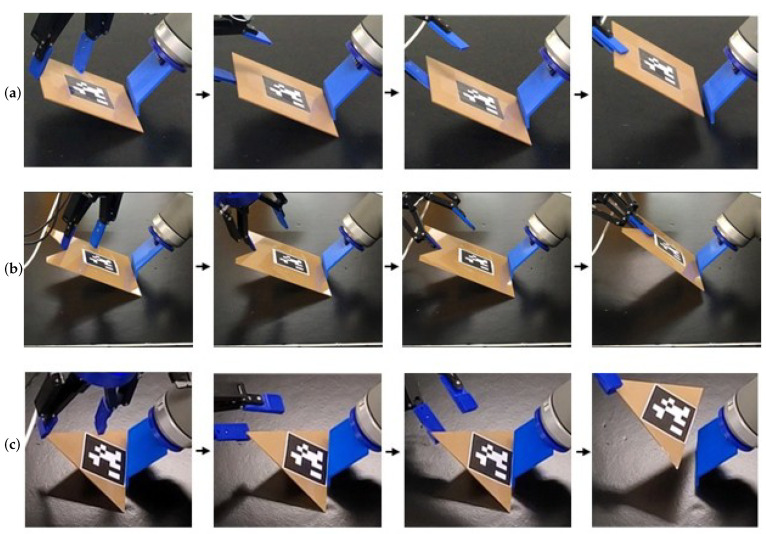
Examples of single-object picking. (**a**) Rectangular acrylic board with a zero Gaussian curvature fingertip. (**b**) Acrylic board with a concave vertex, using a negative Gaussian curvature fingertip. (**c**) Triangular acrylic board with a positive Gaussian curvature fingertip. Reprinted with permission from [1].

For each object, 50 picking trials were conducted using the fingertip with zero Gaussian curvature. A total of 215 successful picks out of 250 trials were achieved, corresponding to a success rate of 86% (see the first row of Table 1, which also lists the target parameters $\left(\psi_{\mathrm{obj}},\psi_{\mathrm{eff}2},\alpha ,\beta ,\gamma \right)$ selected according to Section 4.2). Representative results are illustrated in Figure 8a. Failures were primarily attributed to noisy readings from the force–torque sensor, which occasionally led to incomplete contact formation. In some cases, the arm’s singularities caused the robot controller to halt motion. These limitations could potentially be addressed through additional sensing modalities and singularity-aware motion planning. Importantly, no successful picks were achieved in the absence of fingertip concavity or fingernail design.

Fingertips with nonzero Gaussian curvature were also employed to pick objects by making contact with convex and concave vertices, in addition to edges. See Figure 8b,c. The success rates for these trials remained above 80% (Table 1, rows 2–4). When the robot executed the tilt-and-pivot motion at a higher speed, completing the task in approximately 4 s, the success rate was 80% (row 5 in Table 1). Increasing the force–torque sensing thresholds was found to improve performance. Additionally, trials using a three-fingered gripper (Figure 1) yielded success rates comparable to those obtained with the two-fingered gripper (row 6 in Table 1).

#### 8.2.2. Bin Picking

The method was further evaluated in a practical bin-picking scenario with an increased number of object instances. Multiple acrylic boards were randomly arranged in clutter on a tabletop, and a 2D image of the scene was processed using a Mask R-CNN [33] framework, trained on images of the objects, to achieve instance-level segmentation.

For each picking attempt, the object with the largest visible footprint was selected heuristically, aiming to target the uppermost object. The picking procedure followed the same steps as in the single-object experiments. Prior to each subsequent pick, instance segmentation was performed again to update the scene. This process allowed the cluttered objects to be cleared sequentially, one by one.

**Figure 9:**
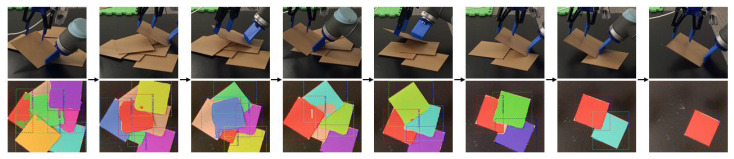
Bin picking of multiple objects using tilt-and-pivot manipulation. Results from instance segmentation are shown alongside the picking sequence. Reprinted with permission from [1].

Table 2 presents the results. For each clutter containing *n* objects, the robot carried out exactly *n* picking attempts, and the experiment was repeated several times. The overall success rate was 203 out of 274 trials, corresponding to 74% (rows 1–5 in Table 2). Figure 9 shows the progress for a clutter of eight objects.

In addition to the failure modes observed in the single-object experiments—particularly noisy force–torque readings—the primary cause of unsuccessful attempts in cluttered settings was inaccurate vision outputs, specifically reduced segmentation accuracy. As the clutter density *n* increases, the performance of instance segmentation degrades (see Figure 9), likely due to significant overlap among object instances. Consequently, the effectors may misalign with the intended target, leading to failed picks—or in some cases, the unintended picking of multiple objects, which is also classified as a failure. This phenomenon explains the observed decrease in success rate with increasing *n* in Table 2.

One way to improve instance segmentation accuracy is to train the vision framework using images with greater object overlap. Furthermore, grasp planning can be re-executed to position the effectors reliably within the interior faces of object instances adjacent to the target, thereby improving instance singulation. Applying this strategy increased the success rate to 47/60, or 78%, as shown by comparing rows 5 and 6 in Table 2.

#### 8.2.3. Beyond Rigid Object Picking

The tilt-and-pivot method can also be applied to flexible objects and extended to more complex manipulation tasks, such as pick-and-assembly, with its implementation on standard robotic hardware enabling smooth integration of these operations.

**Figure 10:**
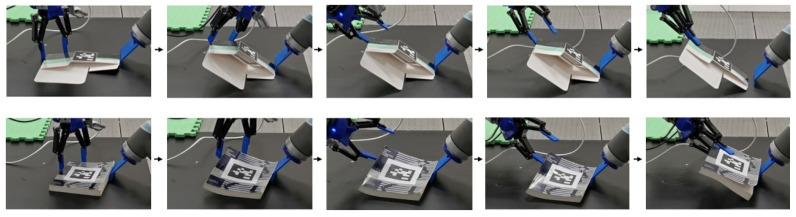
Picking a carton flat (**top**) and a textbook (**bottom**).

**Figure 11:**
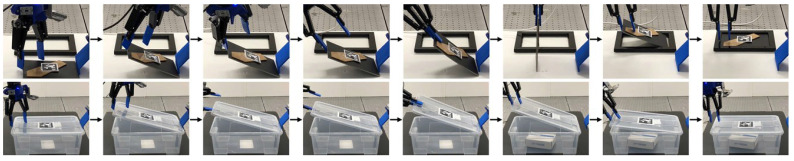
Demonstrations of pick-and-assembly tasks: (**top row**)—picking and assembling a picture frame; (**bottom row**)—opening and closing a container lid. Reprinted with permission from [1].

- Flexible Object Picking: The tilt-and-pivot method can be extended to soft, flexible objects, as demonstrated with the carton flat and paperback textbook (Figure 10 and rows 7–8 in Table 1). During the tilt phase, we arrange the gripper to rotate around an axis nearer to Effector #1 than the contact line at Effector #2, causing the object to bend. In Figure 10, for example, the carton flat accordingly flexes along the internal crease situated between the effectors. This bending helps maintain contact between Effector #1 and the box; without it, contact loss is observed more frequently.
- Pick-and-Assembly: The robot can extend tilt-and-pivot manipulation to more advanced tasks, including picture frame assembly and container lid operation (Figure 11 and rows 9–10 in Table 1). After using tilt-and-pivot to lift the frame backboard or container lid, a shallow-depth insertion operation [18] completes the assembly. In these experiments, regrasping—similar to prehensile pushing [13]—was occasionally required prior to assembly, demonstrating again that secondary operations can be integrated with tilt-and-pivot. The results also indicate that the fingertip concavities or fingernails on the general multi-fingered gripper do not interfere with its other intended functions. In contrast, specialized grippers such as suction grippers may be less effective for these tasks, as they depend heavily on the kinematic architecture or surface conditions of the objects. For example, applying suction to the leg of the frame (the brown part) would cause the frame body to flap downward when lifted.

## 9. Conclusions

In this paper, we introduced tilt-and-pivot, a robotic manipulation technique for picking large, thin objects lying flat on a surface. The method is applicable to a variety of low-profile objects and is not constrained by the workspace size of the gripper. It requires only situational awareness provided by a 2D camera and a wrist force–torque sensor, and can be implemented with standard grippers, making it practical and versatile for real-world applications. Its effectiveness was demonstrated in single-object picking and multi-object bin-picking tasks, highlighting that carefully designed pivoting strategies can enhance grasp reliability even under challenging conditions.

While tilt-and-pivot may involve longer reconfiguration times than fast suction-based approaches and can be sensitive to fingertip geometry, it offers a robust alternative in scenarios where high-speed suction is less effective, such as complex or long-horizon manipulation tasks. Experimental results also indicate that improved situational awareness—achieved through sensor fusion or active perception, a promising direction for future work—can further enhance performance and robustness. Another avenue for future research is the design of reconfigurable grippers to better accommodate variations in object shapes and fingertip concavity, thereby extending the method’s generalizability.

## Figures and Tables

**Table 2:** Experimental results for bin picking. Reprinted with permission from [1].

Clutter Size *n*	Successes/Attempts (n × Repetitions)
8	53/64 (8 × 8)
9	38/45 (9 × 5)
10	43/50 (10 × 5)
11	36/55 (11 × 5)
12	33/60 (12 × 5)
12	47/60 (12 × 5) (with mitigation strategy)

## Data Availability

The original contributions presented in this study are included in the article. Further inquiries can be directed to the corresponding author.
